# An Experimental Approach to Investigate the Involvement of Cognitive Load in Divergent Thinking

**DOI:** 10.3390/jintelligence9010003

**Published:** 2021-01-07

**Authors:** Ruben Kleinkorres, Boris Forthmann, Heinz Holling

**Affiliations:** 1 Center for Research on Education and School Development, TU Dortmund University, 44227 Dortmund, Germany; ruben.kleinkorres@tu-dortmund.de; 2 Institute of Psychology in Education, University of Münster, 48149 Münster, Germany; 3 Institute of Psychology, University of Münster, 48149 Münster, Germany; holling@uni-muenster.de

**Keywords:** divergent thinking, cognitive load, instructions, be-creative effect, serial order effect

## Abstract

Up to now, support for the idea that a controlled component exists in creative thought has mainly been supported by correlational studies; to further shed light on this issue, we employed an experimental approach. We used four alternate uses tasks that differed in instruction type (“be fluent” vs. “be creative”) and concurrent secondary workload (load vs. no load). A total of 51 participants (39 female) went through all tasks and generated ideas for a total of 16 different objects; their responses were scored in terms of fluency (number of responses generated), creative quality, and flexibility. We did find, as expected, that the be-creative instruction resulted in fewer and more creative ideas, as well as more flexible idea sets, but neither of the expected interaction effects became significant. Specifically, fluency was not affected more strongly by secondary workload in the be-fluent instruction condition than in the be-creative instruction condition. Further, the performance drop evoked by the secondary workload was not stronger in the be-creative instruction condition compared to the be-fluent instruction condition when creative quality or flexibility were examined as dependent variable. Altogether, our results do not confirm that be-creative instructions involve more cognitive load than be-fluent instructions. Nevertheless, the analysis of the serial order effect and additional correlational examinations revealed some promising results. Methodological limitations which may have influenced the results are discussed in light of the inherent suspense between internal and external validity (i.e., most likely the applied self-paced dual-task approach increased external validity, but undermined internal validity) and potentially guide future research.

## 1. Introduction

In the past, studies have proposed that the creative process involves a number of sub-processes (Lubart 2001; Mumford and McIntosh 2017), one of which is divergent thinking. The ability to think divergently is characterized by answering a prompt by generating various responses, whereby responses can be either conventional or original (see for example Runco and Acar 2012; Reiter-Palmon et al. 2019). Notably, divergent thinking, as well as convergent thinking, have been suggested to play a crucial role in creative problem solving (Brophy 1998). Yet, since the individual abilities that facilitate divergent thinking are not yet fully understood, contemporary research on this matter has increasingly focused on the underlying cognitive processes.

### 1.1. Different Theories of Creative Cognition

Early on, Mednick (1962) outlined the *associative theory of creativity*, by which he explained differences in creative thinking through individual differences in the hierarchical structure of the semantic network. According to this theory, highly creative individuals show flatter hierarchies than less creative individuals. Furthermore, it suggests that diffuse associative structures allow creative people to more quickly reach remote associations, which in turn may allow them to generate more creative ideas. As such, the associative theory of creativity treats creativity as an effortless bottom-up process. Recently, support was provided for this theory in a study by Kenett et al. (2014), who analyzed the structure of the semantic networks in low- and high-creative persons. In low-creative individuals, they found a more modular and less connected network. Furthermore, they extended Mednick’s (1962) theory by proposing that the network of low-creative individuals is more spread out than that of high-creative individuals. 

A more recent theory is the *controlled-attention theory of creativity*, which emphasizes that creative thinking is a top-down process in which attention is guided by executive control and cognition, resulting in differences in the development of creative ideas (e.g., Beaty et al. 2014; Silvia 2015). In the last decade, the controlled-attention theory of creativity has gained increasing attention. The rather modest correlation reported in Kim’s (2005) meta-analysis between divergent thinking test scores and measures of intelligence (*r* = 0.17) raised the question of whether the relationship had been underestimated, for instance, through the use of outdated statistical methods (Silvia 2008). Subsequently, novel theories, assessment methods, and statistical tools have shown that these constructs, historically viewed as distinct, are more closely connected than previously thought (Silvia 2015). So far, support for the idea that creative thought includes a controlled component has been provided by correlational studies; for example, a substantial relationship between executive functions and performance on divergent thinking tasks has been shown several times through the use of latent variable models (see for example Süß et al. 2002; Nusbaum and Silvia 2011; Benedek et al. 2012a; Silvia et al. 2013; Benedek et al. 2014b; Pan and Yu 2016). Moreover, neuroimaging studies provide evidence that the creativity of ideas in divergent thinking tasks is related to the activation of brain regions that are associated with executive functioning, thus also supporting the idea that executive processes are involved in divergent thinking (see for example Benedek et al. 2014a).

Finally, some studies have shown that creativity involves both associative abilities as well as cognitive processes (e.g., Beaty et al. 2014). Recently, Beaty et al. (2019) and Forthmann et al. (2016) put forward studies that assessed the components of creativity using an experimental approach: Upon manipulating the type of instruction or/and the frequency in which task stimuli occurred in language, they found that divergent thinking involves both associative bottom-up processing and executive top-down processing. Relatedly, it has been proposed that creative thinking requires flexible cognitive control that allows shifting between defocused and focused attentional states (Gabora 2010; Zabelina et al. 2016a; Zabelina 2018). Zabelina et al. (2016a) found that better divergent thinkers display flexible attention that allows quick overcoming of irrelevant information. Another study by Zabelina et al. (2016b) revealed a more nuanced pattern and found that divergent thinking requires either a medium level of cognitive control in combination with cognitive flexibility or very strong reliance on cognitive control in case that flexibility is low. Hence, flexibility plays a vital role in creative thinking. This is further highlighted in the neurocognitive framework proposed by Zhang et al. (2020). They combine the dual pathway theory of creativity (Nijstad et al. 2010) with Hommel’s Metacontrol State Model (Hommel 2015) as a rather general cognitive control model. For divergent thinking, it is proposed that flexibly switching between conceptual categories facilitates creative thinking, for example. For convergent thought (Cropley 2006), however, the persistence route is considered to be more important. Zhang et al.’s model allows conceptualizing varying degrees of compositions of flexibility and persistence that can explain the findings mentioned above by Zabelina et al. (2016a) or Zabelina et al. (2016b). In addition, this idea is critically important to the current work when different instructions for divergent thinking are compared.

### 1.2. The Be-Creative Effect: Support for the Controlled-Attention Theory?

The standard instruction (Runco and Acar 2010) used in divergent thinking tasks asks participants to generate as many ideas as possible for the task stimuli. For example, in the alternate uses task (AUT; Wallach and Kogan 1965)—a frequently used divergent thinking task—participants are asked to name as many uses as possible for a given object (e.g., “Tell me all the different ways you could use a knife”; Wallach and Kogan 1965, p. 31). However, this “be fluent” instruction has been increasingly questioned: Some authors have indicated that it might lead different participants to assume different task goals, as participants might not be sure whether they should be productive or be creative (e.g., Harrington 1975; Nusbaum et al. 2014). Moreover, Nusbaum et al. (2014) reiterated the point made by Carroll (1993, p. 429) and suggested that the standard be-fluent instruction means that divergent thinking tasks strongly resemble verbal fluency tasks, especially when ideas are scored in terms of fluency. As such, they argued that researchers using this type of instruction would be measuring verbal fluency rather than divergent thinking.

Studies on the controlled-attention theory of creativity usually rely on instructions that focus on being creative. Researchers advocating this theory argue that the widespread use of the standard be-fluent instruction is partially responsible for the rather modest relationship observed between intelligence and creativity (Benedek et al. 2014b; Nusbaum et al. 2014). Several studies have shown that the explicit instruction to be creative raises the creative quality of ideas while reducing fluency, and, therefore, enhances the validity of divergent thinking tasks (Harrington 1975; Runco and Okuda 1991; Nusbaum et al. 2014). This finding is called the be-creative effect (e.g., Forthmann et al. 2016). Furthermore, some authors have indicated that divergent thinking relies more strongly on executive control when the instructions are to be creative than when they are to be fluent (standard instructions): By using a multilevel latent variable model, Nusbaum et al. (2014) showed that when the be-creative instruction was used, the creative quality of ideas within an AUT was predicted by fluid intelligence (i.e., the reasoning and problem-solving component of executive functions; see Diamond 2013). In contrast, when the be-fluent instruction was used, fluid intelligence did not significantly predict fluency. Guilford (1968) further argued that be-creative instructions might involve evaluative thinking more strongly and, hence, the composition of flexibility and persistence might shift towards a metacontrol bias (Zhang et al. 2020).

However, at the same time, verbal fluency tasks—which seem to be similar to divergent thinking tasks that use the be-fluent instructions, as mentioned above—are still considered to involve strategic memory retrieval. For example, Rosen and Engle (1997) compared individuals with different working memory capacities and found that they performed differently on a category-generation task when a concurrent “interfering” task was present. In the group of individuals who had a high working memory capacity, the interfering task led to a stronger decrease of fluency scores compared to those in the group with a low working memory capacity. Furthermore, verbal fluency tasks are frequently used as measures for executive functioning (e.g., Alvarez and Emory 2006) and are even used as indicators of executive ability in divergent thinking research (e.g., Beaty et al. 2014; Gilhooly et al. 2007). Assuming that divergent thinking tasks with a be-fluent instruction are similar to verbal fluency tasks, the question arises as to which type of instruction leads to thinking processes that rely more strongly on cognitive control.

### 1.3. The Present Study

In the present study, we wanted to provide an experimental approach to address this issue. Similar to the study by Rosen and Engle (1997), we manipulated the workload under which participants had to complete another task; however, we applied this paradigm to an AUT instead of a verbal fluency task. Additionally, we varied the type of instruction. In line with the controlled-attention theory, our aim was to show that instructions to be creative involve more cognitive control than instructions to be fluent. Thus, the following five hypotheses were formulated: 

In line with previous findings (Nusbaum et al. 2014; Forthmann et al. 2016), we expected that the be-fluent instruction should lead to a higher fluency score than the be-creative instruction, at least in the no-load condition. Further, we expected an overall main effect of instruction type on fluency to be driven by the difference for the no-load condition (H1). Furthermore, we sought to replicate the other component associated with the be-creative effect. Therefore, the be-creative instruction should lead to a higher creative quality of ideas than the be-fluent instruction, at least in the no-load condition (see Nusbaum et al. 2014; Forthmann et al. 2016), and we expected an overall main effect of instruction type on creative quality to be driven by the difference for the no-load condition (H2). In accordance with the idea that be-creative instructions involve more cognitive control as compared to be-fluent instructions, we further expected that flexibility would be higher for be-creative instructions than in the be-fluent instruction (H2b). Again this was expected at least for the no-load condition, as it has been demonstrated by Forthmann et al. (2018).

Moreover, we assumed an interaction effect between workload (load vs. no load) and instruction type (be fluent vs. be creative) such that the performance drop concerning quantity (i.e., fluency) evoked by a concurrent task would be stronger in the be-fluent instruction condition compared to the be-creative instruction condition (H3). More precisely, we expected that workload would have no effect on fluency in the be-creative instruction condition because of the assumption that cognitive resources such as controlled attention are used to generate ideas high in quality instead of sheer quantity (i.e., instruction-scoring fit; see Reiter-Palmon et al. 2019). Although we did not rule out the possibility that an overall main effect for workload would be driven by its impact on fluency in the be-fluent instruction condition, we did not explicitly assume that effect. On the other hand, the performance drop concerning creative quality evoked by a concurrent task should be stronger in the be-creative instruction condition compared to the be-fluent instruction condition (H4), because the be-fluent instruction mainly applies cognitive resources such as controlled attention to the quantity of ideas generated instead of the creative quality, thus sparing any performance drop on creative quality. Similar to the assumption posited in the preceding hypothesis, we did consider but not expect an overall main effect for workload to be driven by its impact on creative quality in the be-creative instruction condition. Finally, and most importantly, we expected the interaction effect regarding creative quality (H4) to be more strongly pronounced than the interaction effect regarding quantity (H3). Ultimately, these findings would support the notion that instructions to be creative involve more cognitive control compared to instructions that demand the generation of as many ideas as possible (H5).

## 2. Methods and Materials

### 2.1. Preregistration

This study was preregistered, and the preregistration file including the study plan and planned analyses can be found in an Open Science Framework (OSF) repository: https://osf.io/ajhgc/.

### 2.2. Power Analysis

The effects found by Rosen and Engle (1997) formed the basis for estimating the sample size needed in the present study. In their experiment, they found a large main effect of secondary workload (load vs. no load) on fluency achieved in a category-generation task (Cohen’s *d* = 0.82, 95%-CI: [0.31, 1.33]). Because verbal fluency tasks resemble divergent thinking tasks that have a be-fluent instruction, we expected this effect for the fluency performance drop in this instruction condition. Moreover, as we did not expect a fluency performance drop in the be-creative instruction condition, this effect was also presumed for the interaction effect regarding fluency as the dependent variable. Furthermore, Rosen and Engle (1997) found an even stronger effect of workload on fluency for individuals with high working memory capacity (Cohen’s *d* = 1.39, 95%-CI: [0.64, 2.14]); as explained by the researchers, this stronger performance drop occurred because these individuals involved working memory to a greater extent than those with lower working memory capacity. In the present study, however, instead of using individuals with differences in working memory capacity, we expected a stronger working memory load to be evoked according to the explicit instruction. Thus, this effect was presumed for the interaction effect with creative quality as the dependent variable, since the secondary workload should only evoke a quality performance drop in the be-creative instruction condition. Certainly, the effects found by Rosen and Engle (1997) cannot be transferred perfectly to the effects we expected with our study design, but the effects reported in that study are, to our knowledge, the best estimation available in the literature.

Taken together, for our fifth hypothesis, we expected a difference in standardized measurement units of 0.57. To compute the required sample size for the one-sided *t*-test of the difference of both effect sizes, we used the statistical software R (R Core Team 2014). Besides an assumed alpha level of 5% and a desired power of 80%, we expected—grounded on correlational results in prior studies (e.g., Forthmann et al. 2017b)—an average correlation between both dependent variables within the same instruction condition of *r* = 0.40. Based on 100,000 replications, a sample size of *n* = 47 was projected to achieve the desired level of power. 

We increased the sample size to address the following exclusion criteria: Analogous to Rosen and Engle (1997), we intended to exclude participants who missed more than 50% of the critical digits during the digit tracking. Since approximately 3% of participants had to be removed in their study, we expected two participants to meet the exclusion criterion in our study. This led to an adjusted sample size of *n* = 49. Furthermore, we planned to exclude those who failed to complete the AUT properly (e.g., participants who repeatedly gave nonsensical answers). Because such cases are difficult to predict, we decided to conservatively expect that 15% of participants would repeatedly give nonsensical answers or something similar. This led to an adjusted sample size of *N* = 56. 

### 2.3. Participants

According to the results of the power analysis, 56 participants were tested. One participant was excluded because the instructions were not properly followed. Two more participants gave a large number of nonsensical answers and were also excluded. Furthermore, the data of two participants were removed because they missed 50% of the critical digits. Thus, our final sample consisted of *n* = 51 participants (39 female). The age ranged from 18 to 38 years (*M* = 23.55, *SD* = 4.32). The sample was composed of 49 undergraduates and two employees. Undergraduates from the subject areas psychology and sports science signed up for the experiment in exchange for course credit.

### 2.4. Materials

The computer software Inquisit 4 (http://www.millisecond.com) was used to present the tasks and collect responses.

#### 2.4.1. Divergent Thinking Tasks

Participants completed four AUTs each containing four objects. Because we wanted to prevent carryover effects evoked by semantic similarities between objects within a single AUT, and because performance on the tasks should not be influenced by the frequency of stimuli, which can affect the amount of associations (see Forthmann et al. 2016), we chose 16 objects from four underlying categories (furniture, vegetable, tool, musical instrument). Here, the four objects of each category were matched—as much as possible—to each other in terms of lemma frequency (see Appendix A, Table A1). The lemma frequency indicates the number of occurrences of all the inflectional variants of a particular word (see for example Bien et al. 2005). The object selection in accordance with the corresponding lemma frequencies was facilitated through a database provided by Schröder et al. (2012). 

In our experiment, objects were assigned to the AUTs in a computerized manner. A single AUT was constructed by selecting one object randomly from each category, after which that object was not available to be selected again. Thus, at the end of the experiment, each object had been assigned to one of the tasks. Importantly, the resulting tasks consisted of a similar pool of object categories and lemma frequencies, which should reduce the influence of object characteristics on task performance. 

The procedure for a single AUT was as follows: Throughout the entire working period, objects were presented sequentially, and the display duration depended on the participant’s response time; the maximum display duration per object was 50 s. To prevent sequence effects, the order of the objects was randomized, whereby the objects that had been assigned to the task were selected randomly without replacement. Therefore, each time a sequence of four objects was completed, a new random sequence of the same objects started. The participant’s response time for each object was recorded, and the task ended when the sum of response times exceeded the time limit of 10 min.^1^ The participant was then allowed to complete his last idea. 

According to previous studies (e.g., Harrington 1975; Nusbaum et al. 2014; Forthmann et al. 2016), we used two different types of instructions for our AUTs. In two of the four experimental tasks, we gave participants the standard be-fluent instruction, which simply asked them to name as many uses as possible for the objects presented. In the other two tasks, we gave them the be-creative instruction, which asked them for as unusual uses as possible for the given objects. To clarify the task procedure, three examples were added to the different instruction types (for detailed instructions, see Appendix B, Appendix C, Appendix D, Appendix E, Appendix F).

#### 2.4.2. Digit-Tracking Task

We varied the workload under which participants completed the AUTs by implementing a concurrent task in half of the experimental tasks. According to Rosen and Engle (1997), we used the digit-tracking task.^2^ In our study, the task was embedded in the AUTs in the following way: Each time a participant gave a response for the most recently presented object, a digit 1–9 appeared for 1 s in a corner of the screen. The first digit in an experimental block was displayed in the upper left corner; the following digits then appeared clockwise in the other corners of the screen. Whenever a participant saw a third consecutive odd digit appearing in the sequence, he was instructed to press the space bar of the computer keyboard. Depending on the participant’s response, visual feedback (“Correct” vs. “Wrong”) was provided for a short period (1 s) in the center of the computer screen. Another instance of feedback appeared if the participant missed three or more events^3^ in a row; he was then reminded to pay more attention to the digit-tracking task (see Rosen and Engle 1997). This reminder feedback was displayed for 7 s in the center of the computer screen. There was no fixed number of events, but the probability of an odd digit appearing was set to 75%, making the probability that three odd digits appeared in a row about 42% (0.75 × 0.75 × 0.75 = 0.42). However, if an event occurred, the probability that the next digit was odd was 0%; thus, the actual probability of three odd digits in a row was lower than 42%. This rule was implemented to prevent the occurrence of four or more odd digits in a row, which would have been confusing for participants.

#### 2.4.3. Ideational Behavior

The common procedure of AUTs requires participants to work on a single object for a fixed time before continuing with the next object (see, for example, Silvia et al. 2009; Nusbaum et al. 2014; Forthmann et al. 2016). However, as mentioned above, we presented the objects in a random sequence, and we wanted to check whether this procedure was comparable with the common AUT procedure. Since AUTs are related to self-reported ideational behavior, we used the German version (see Benedek et al. 2012b) of the Runco Ideational Behavior Scale (RIBS; Runco et al. 2001) to determine the criterion validity of our experimental design. The German version of the RIBS contains 17 items, each describing actual behaviors that reflect an individual’s use of ideas; the RIBS can be seen as a criterion of creative ideation (Runco et al. 2001). Reliability for the sample in this study was estimated to be good, Cronbach’s α = 0.87.

### 2.5. Procedure

The experiment was conducted primarily using a computer and took about 65 min. Participants were tested in groups of up to seven people. After completing the RIBS, which was handed out as a paper-and-pencil questionnaire, participants’ typing speed was assessed by asking them to type the names of all calendar months as fast as they could in given text boxes within 1 min (for a detailed instruction see Appendix G). This was done to control for the influence of typing speed on the dependent variables (see Forthmann et al. 2017b). Then, participants went through three practice trials (single AUT, single digit-tracking task, AUT and concurrent digit-tracking task) to get familiar with the different task formats^4^ (for detailed instructions see Appendix H, Appendix I, Appendix J, Appendix K). If participants made more than one mistake in one of the practice trials, which included the digit-tracking task, the corresponding task was repeated.^5^ Subsequently, the actual experiment started, which consisted of four experimental tasks considering all factor level combinations (two instruction types × two load conditions). Participants with an odd running number started with the two single AUTs without the secondary workload and continued with the combined tasks comprising AUT and concurrent digit tracking. Participants with an even running number performed the task groups in the opposite order. Additionally, the order of instruction type was randomized. As a result, our experiment consisted of eight different running orders (see Appendix L). At the end of the experiment, participants were told to indicate whether they used their fingers to assist during the digit-tracking task (e.g., to count the number of odd digits appearing in a row). This was done to receive information about the influence of strategy use on task performance.

### 2.6. Scoring Divergent Thinking Tasks

Before the actual scoring of the divergent thinking tasks started, we prepared data by sorting out nonsensical answers and duplicate ideas (i.e., the criteria used for response adequacy; see Reiter-Palmon et al. 2019). Afterwards, participants’ ideas were scored in terms of quantity (i.e., fluency) and creative quality. Fluency scores were built by simply counting the number of ideas per participant and AUT. For creative quality, scores were obtained through subjective ratings of creative quality (see Silvia et al. 2008; Forthmann et al. 2017a), for which three judges were involved. To derive a single creative quality rating for each idea, the judges used the three scoring dimensions uncommonness, remoteness, and cleverness defined by Wilson et al. (1953), as well as the additional scoring dimension appropriateness, which points to the applicability, the general practicability, and the usefulness of an idea. According to prior studies (see Silvia et al. 2008; Forthmann et al. 2016), a 5-point rating scale (ranging from 1 = low quality to 5 = high quality) was used to assess the degree of creative quality. After the ratings were completed, the data was revised for a second time by removing ideas that had been assessed by at least two of three raters as “nonsense”. To raise the reliability of the ratings, we used the average of the raters’ scores for each idea. Inter-rater reliability for the total set of ideas^6^ was ICC(2,1) = 0.661, 95%-CI: [0.622, 0.695] for the single ratings and ICC(2,3) = 0.854, 95%-CI: [0.829, 0.874] for the average scores. The calculation of the ICC was based on absolute agreement.

Next, flexibility was scored according to a set of 21 categories used by Forthmann et al. (2018). Please note that flexibility scoring was added during the process of revising this work and, hence, this scoring was not included in the preregistered analyses plan. In the case that a response could not be assigned to one of the categories, it was assigned to a unique category. The first and second author of this work acted as raters for flexibility scoring. Both are experienced raters of divergent thinking responses (for both creative quality and flexibility). Responses were pre-categorized prior to scoring by two student assistants. That is, synonymous responses were cross-tabulated as it is commonly done for frequency-based originality scoring (Forthmann et al. 2020b; Reiter-Palmon et al. 2019). This step facilitated flexibility scoring, because only non-redundant responses had to be rated. To reactivate the necessary frame of reference for scoring, both raters categorized a random sample of 100 responses and achieved a Krippendorff’s α of 0.66 for nominally scaled coding variables. This inter-rater reliability was not considered sufficient. Hence, after discussing differences between the ratings of both coders on the initial set of responses, a second round of 100 randomly chosen responses was coded. Inter-rater reliability was considered sufficient after this second round of coding (Krippendorff’s α = 0.78). Then, each rater coded all non-redundant responses that were initially cross-tabulated for eight of the objects. After merging the flexibility data with all other relevant data, it became apparent that a total of 948 responses did not receive a category during the rating process. These responses were then scored by the second author of this work. 

Creative quality scores were aggregated per AUT and for each participant. Because using the mean as a participant’s creative quality score may be biased by the least creative ideas, and using the maximum omits relevant information, as was argued by Forthmann et al. (2017a, 2017b), we approached a more valid measure for the creative outcome: we used the 75% quantile as an indicator of a participant’s overall creative achievement. This approach was preregistered. In summary, each participant ended up with eight scores (four fluency scores and four creativity scores).

Flexibility categories were also aggregated per AUT and for each participant. However, flexibility scoring for the multi-object DT paradigm as used in this work is not straightforward. That is, we opted for a clever binary scoring of category switches (Acar and Runco 2017; Acar et al. 2019) that prevents the fluency confounding when an appropriate aggregation method is used (Forthmann et al. 2020a). However, given that objects alternate from response to response in the multi-object paradigm, category switches from response to response are too likely and are expected to have a quite low variation. Hence, we used a coding that was still inspired by this approach and coded a response as one when the category it was assigned to did not occur for each of the previously generated responses, whereas a score of zero was assigned for each response that was associated with a category that was used before. We averaged this scoring at the level of persons for each of the AUT conditions or analyzed this flexibility score directly at the level of responses based on multilevel logistic models (see Acar et al. 2019).

### 2.7. Analytical Approach

Data analysis was conducted using the statistical software R (R Core Team 2014). All analyses scripts and data files needed to reproduce the reported analyses are openly available in an OSF repository: https://osf.io/ajhgc/.

#### 2.7.1. Testing of Hypotheses

To test our first two hypotheses, we conducted two one-sided paired-sample *t*-tests. Further, to test the interaction effects stated in Hypotheses 3 and 4 as well as the overall main effects stated in Hypotheses 1 and 2, we performed two repeated-measures analyses of variance with fluency and creative quality as dependent variables, respectively. The R package *ez* (Lawrence 2013) was used to calculate the ANOVAs. The variables typing speed and usage of fingers as help (yes vs. no) did not seem to influence the results of the ANOVAs and were therefore not considered. Finally, we conducted a one-sided paired-sample *t*-test to test hypothesis 5. The rntransform() function of the R package GenABEL (GenABEL project developers 2013) was used to transform both outcomes (fluency and creative quality) on the same scale, making these outcomes comparable.

#### 2.7.2. Validation of Divergent Thinking Task Procedure

Criterion validity of our experimental design was determined by calculating—separately for each experimental condition—correlations between average scores achieved in the RIBS (Runco et al. 2001) and fluency scores as well as scores for creative quality. For further validation of the divergent thinking task procedure, we tested a typical finding in divergent thinking research called the serial order effect (Christensen et al. 1957); this effect is characterized by an increase in the creativity of ideas over time during the working period (Christensen et al. 1957). Similar to Beaty and Silvia (2012), we segmented time on task into ten 1-min intervals. By means of the R package nlme (Pinheiro et al. 2015), a linear mixed-effects model was calculated to estimate the influence of time on creative quality of ideas. For flexibility as it was scored in this work, we fitted a logistic mixed-effects model by means of the R package lme4 (Bates et al. 2015). In line with former analyses of the serial order effect (e.g., Beaty and Silvia 2012), we entered a linear and a quadratic term of time as fixed effects into the model. As random effects, we had intercepts for participants. Again, typing speed and usage of fingers did not seem to influence the dependent variable and were therefore not considered. 

#### 2.7.3. Exploratory Analyses 

In the last step of our analysis, we further explored the serial order effect (for creative quality and flexibility) and inspected correlations between the dependent variables and several measures relating to the digit-tracking task. These analyses were not preregistered.

- **Serial order effect:** In addition to the linear mixed-effects model that included only effects of time as predictors, we explored the changes in model fit that occurred when we considered the factors workload and instruction. To track differences between conditions over time, various combinations of additive and interactive terms of both predictors were added as fixed effects into further models. As in the above analysis, intercepts for participants were the random effects.
- **Correlations:** Finally, we explored correlations between fluency, creative quality, dose (i.e., the number of digits presented during the AUTs in the load condition), accuracy (i.e., the percentage of correct reactions on an event within the digit-tracking task), and RIBS scores. The correlations were calculated separately for each experimental condition.

## 3. Results

### 3.1. Testing of Hypotheses

Table 1 displays mean fluency and quality scores separately for all four different factor level combinations. Fluency scores were clearly higher in the be-fluent instruction condition than in the be-creative instruction condition, while the opposite was true for creative quality scores. At first sight, workload does not seem to have evoked any differences in fluency or creative quality.

The first one-sided paired-sample *t*-test showed that, for the no-load condition, the be-fluent instruction led to a significantly higher fluency score than the be-creative instruction (see Table 1), *t*(50) = 11.364, *p* < 0.001, *d* =1.571.^7^ Furthermore, the repeated-measures ANOVA with fluency as the dependent variable revealed a significant overall main effect of instruction type on fluency, *F*(1,50) = 140.157, *p* < 0.001, η_p_^2^ = 0.364. The second one-sided paired-sample *t*-test showed that, for the no-load condition, the be-creative instruction led to a significantly higher score of creative quality than the be-fluent instruction (see Table 1), *t*(50) = 10.722, *p* < 0.001, *d* =1.788. Furthermore, the repeated-measures ANOVA with creative quality as the dependent variable revealed a significant overall main effect of instruction type on creative quality, *F*(1,50) = 165.113, *p* < 0.001, η_p_^2^ = 0.470. Taken together, these results confirmed our first two hypotheses and, therefore, the be-creative effect. 

The repeated-measures ANOVA with fluency as the dependent variable did not show a significant interaction between the manipulated factors workload and instruction type, *F*(1,50) = 0.862, *p* = 0.358, η_p_^2^ = 0.001. Moreover, no effect of workload on fluency was apparent,^8^ *F*(1,50) = 1.172, *p* = 0.284, η_p_^2^ = 0.001. The repeated-measures ANOVA with creative quality as the dependent variable revealed similar results: No significant interaction was observed between the manipulated factors workload and instruction type,^9^ *F*(1,50) = 0.141, *p* = 0.709, η_p_^2^ = 0.000, nor was there an apparent effect of workload on creative quality, *F*(1,50) = 0.218, *p* = 0.643, η_p_^2^ = 0.000. Since neither of the interaction effects became significant, no further comparisons were conducted. Therefore, Hypotheses 3, 4, and 5 could not be confirmed.

In addition, a one-sided paired-sample *t*-test performed in the no-load condition displayed a significantly higher score of flexibility in the be-creative than the be-fluent instruction (see Table 1), *t*(50) = 10.113, *p* < 0.001, *d* =1.763. The difference between both instruction types was generally reliable as revealed by a significant main effect in the repeated measures ANOVA with flexibility as dependent variable, *F*(1,50) = 146.081, *p* < 0.001, η_p_^2^ = 0.399. Workload did not have a main effect on flexibility, *F*(1,50) = 0.950, *p* = 0.335, η_p_^2^ = 0.002. The instruction × workload interaction was marginally significant, *F*(1,50) = 4.014, *p* = 0.051, η_p_^2^ = 0.009. A one-sided contrast between both be-creative instructions further revealed that the be-creative condition yielded significantly more flexible responses as compared to the be-creative + workload condition, *t*(50) = 1.991, *p* = 0.026, *d* = 0.237. Workload did not display this difference for the be-fluent instruction condition, *t*(50) = −0.759, *p* = 0.774, *d* = −0.124. 

### 3.2. Validation of Divergent Thinking Task Procedure

**Figure 1:**
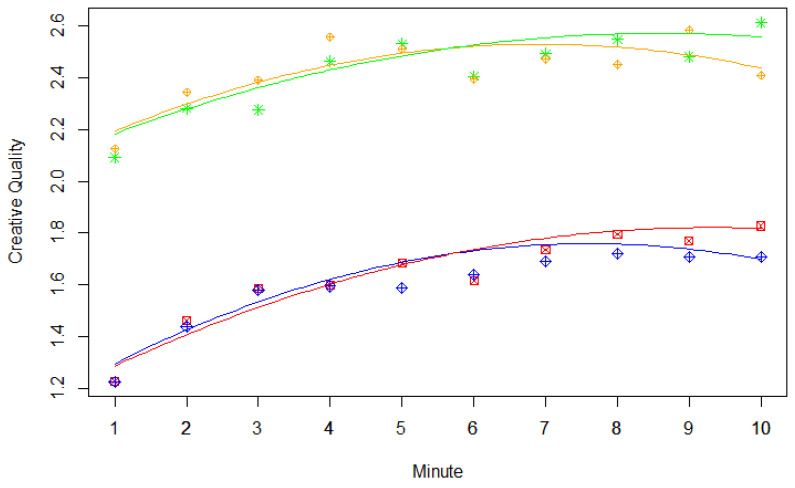
Creativity of divergent thinking responses across time depending on instruction type and load condition. Regression lines are based on the regression weights for the fixed effects obtained from Model 8. Single points indicate creativity ratings averaged over participants. Green stars = no load + be-creative instruction, orange rhombuses = load + be-creative instruction, red squares = no load + be-fluent instruction, and blue rhombuses = load + be-fluent instruction.

In the first step of validating our experimental design, we determined criterion validity. However, the correlations between RIBS scores and the dependent variables were very low and, in some cases, even negative (see Table 4). Therefore, these correlations could not support the validity of our experimental design. In a next step, we sought to replicate the serial order effect. Regression weights and model comparison statistics of different linear mixed-effects models investigating the serial order effect can be seen in Table 2. In line with previous findings (see, for example, Beaty and Silvia 2012), we found significant linear and quadratic effects of time on the creative quality of ideas, whereby linear effects were positive and quadratic effects were negative (see Table 2). As shown in Figure 1, the increase in creative quality over time was apparent in each experimental condition. Furthermore, the quadratic effect of time is also visible, as creativity reaches a kind of plateau at the end of the time period (see Figure 1). As mentioned above, the serial order effect is a typical finding in research on divergent thinking, and, therefore, its occurrence can be considered evidence for the validity of our experimental task procedure.

### 3.3. Exploratory Analyses

#### 3.3.1. Serial Order Effect—Creative Quality

When comparing Model 4 and Model 1, we see a large effect of instruction on creative quality, while load did not significantly enhance the model fit (see Table 2). These results are not very surprising, as they correspond to those observed in the ANOVA with creative quality as the dependent variable. 

Furthermore, simultaneously considering the interaction of workload with the linear effect of time and the interaction of workload with the quadratic effect of time improved model fit, as is seen in the comparison between Model 7 and Model 6 (see Table 2). Although neither of these interaction terms significantly predicted creative quality, the effect for the interaction of workload with the quadratic effect of time was far greater than the interaction of workload with the linear effect of time, as shown by the ratio between regression weight and standard error (see Table 2). In Figure 1, this effect is apparent in the slight decrease in creative quality at the end of the time period when the load condition was used instead of the no-load condition (orange and blue graphs). 

Simultaneously considering the interactions of instruction with the linear and quadratic effects of time, respectively, led to comparably inconclusive results: Neither of the added predictors significantly influenced creative quality, but their collective influence enhanced the model fit. This finding was apparent in the comparison between Model 8 and Model 7; here, the effect regarding the interaction of instruction with the linear effect of time was much greater than the interaction of instruction with the quadratic effect of time (see Table 2). With a view to Figure 1, this effect can be seen by the steeper increase of creative quality across time when the be-fluent instruction condition was used (red and blue graphs).

According to the results described above, Model 8 seems to be the most appropriate model, as it considered interactions of the manipulated factors with both time effects. However, when the fit indices AIC and BIC were consulted as indicators for model fit, results were inconclusive: Comparison of AIC values confirmed Model 8 as the best-fitting model, while comparison of BIC values revealed that Model 4 was the most appropriate model.

Further analysis revealed that the interaction of instruction with the linear effect of time (β = 0.018, *p* < 0.001) significantly predicted creative quality when the interaction of instruction with the quadratic effect of time was removed from Model 8. Moreover, comparison with Model 8 revealed a better model fit (AIC = 16,788.68, BIC = 16,858.62). Taken together, weak evidence suggests that the factor instruction affects the linear effect of time on creative quality, while load influences the quadratic effect of time on creative quality.

#### 3.3.2. Serial Order Effect—Flexibility

**Figure 2:**
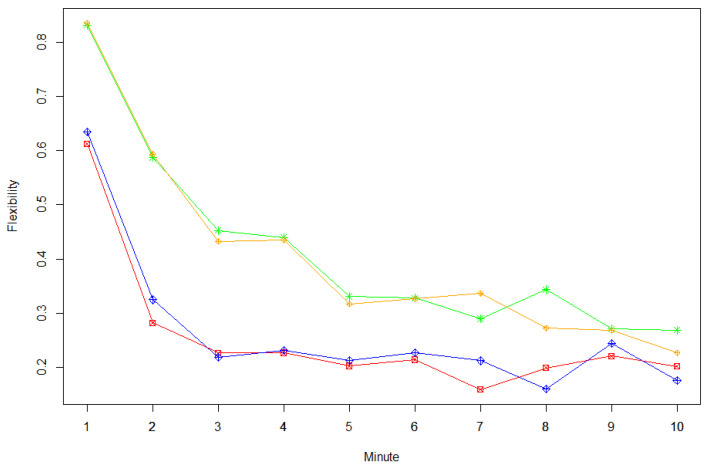
Flexibility of divergent thinking responses across time depending on instruction type and load condition. Single points indicate the relative frequency of responses that were generated from a category that was not used before (i.e., aggregated over participants for each experimental condition). Green stars = no load + be-creative instruction, orange rhombuses = load + be-creative instruction, red squares = no load + be-fluent instruction, blue rhombuses = load + be-fluent instruction.

Again, when comparing Model 4 and Model 1, we see a large effect of instruction on flexibility, while load did not significantly enhance the model fit (see Table 3). Furthermore, for flexibility, only the interaction-term for time and instruction (Model 5) further improved model fit beyond Model 4 (see Table 3). In Figure 2, this effect is apparent as a steeper decreasing slope at earlier time intervals of the relative frequency for a response out of an unused category for the be-fluent (red and blue graphs) vs. be-creative instructions (orange and green graphs). Model 5 was also clearly the best fitting model with respect to all examined criteria (see Table 3).

#### 3.3.3. Correlations

One of the most obvious findings of the exploratory analysis concerning correlations between the dependent variables fluency, creative quality, and flexibility, as well as dose and accuracy, was the high positive correlation between dose and fluency independent of the experimental condition (see Table 4). This result was to be expected, since—according to our experimental design—the number of presented digits increased with the number of ideas given by a participant. In contrast, dose and creative quality correlated negatively. As these negative correlations were also discernible in the no-load conditions, it seemed unlikely that creative quality was directly affected by the number of digits presented within an experimental block (the difference between correlations was indeed non-significant: *z* = 0.538, *p* = 0.591). However, for flexibility, the pattern of correlations was more in line with the idea of direct effect of dose on creative thinking. The negative correlation between dose and flexibility was stronger for the be-creative+load condition (*r* = −0.647) as compared to the be-fluent+load condition (*r* = −0.451), but here also the difference was non-significant (*z* = −1.53, *p* = 0.126). 

Due to the negative correlations between fluency (which also correlated positively with dose, as mentioned above) and creative quality, it seemed further possible that the relationship between dose and creative quality (and flexibility) was mediated by fluency. However, when fluency was partialled out and the load condition was considered, the partial correlation between dose and creative quality in the be-creative instruction+load condition remained significant (*r*_Dose BC, Quality BC (load)_ = −0.389, *p* = 0.003), whereas the partial correlation between dose and creative quality in the be-fluent instruction+load condition did not (*r*_Dose BF, Quality BF (load)_ = −0.252, *p* = 0.071). However, the difference between these two partial correlations as indicated by Steiger’s test (Steiger 1980) was non-significant, *z* = −0.86, *p* = 0.39. For flexibility, the negative correlation between dose and flexibility did not persist in the be-creative instruction+load condition after statistical control of fluency (*r*_Dose BC, Flexibility BC (load)_ = −0.253, *p* = 0.070), whereas this correlation was also non-significant for the be-fluent instruction+load condition (*r*_Dose BF, Quality BF (load)_ = 0.135, *p* = 0.347). The difference between these correlations was, however, found to be significant, *z* = −2.10, *p* = 0.036. 

Looking at Table 5, we see a surprisingly high correlation between the doses in both instruction conditions. Since dose and fluency were strongly correlated, we thought that the correlation between the doses was mediated by the number of ideas given. However, if we partial out fluency scores in the load-condition with a be-fluent instruction and fluency scores in the load-condition with a be-creative instruction, respectively, from the doses in the corresponding experimental conditions, the relationship between the doses did not disappear. Instead, the correlation between the variables was even higher (*r* = 0.610, *p* < 0.001). Another noteworthy finding in Table 5 is that the number of presented digits correlated positively with the accuracy in the corresponding AUT. Further analysis, however, revealed that the correlation between dose and accuracy was only significant in the be-creative instruction condition (*r* = 0.357, *p* < 0.05).

## 4. Discussion

Despite the many studies that have investigated controlled-attention theory (see for example Süß et al. 2002; Nusbaum and Silvia 2011; Benedek et al. 2012a; Silvia et al. 2013; Benedek et al. 2014b; Pan and Yu 2016), the experimental evidence for a controlled component in creative thought is quite weak. In the present study, a digit-tracking task was implemented to manipulate the workload under which participants had to perform divergent thinking tasks. Additionally, the instruction type was varied. This experimental approach was used to show that cognitive load is more strongly involved when people are asked to be creative than when they are asked to be prolific (to generate many ideas).

We were able to show that the explicit instruction to be creative raises the creative quality of ideas and the flexibility of the responses, while reducing fluency. Therefore, we were able to replicate the be-creative effect, which has been shown by previous research (e.g., Nusbaum et al. 2014; Forthmann et al. 2016). In addition, an advantage with respect to flexibility for be-creative instructions has been replicated (Forthmann et al. 2018). However, we did not find either of the expected interaction effects: Fluency was not affected more strongly by workload in the be-fluent instruction condition than in the be-creative instruction condition. Moreover, the performance drop evoked by workload was not stronger in the be-creative instruction condition compared to the be-fluent instruction condition when creative quality was the dependent variable. Consequently, we could not confirm the most important assumption of the present study, which was that instructions to be creative involve more cognitive control than instructions demanding as many ideas as possible. 

### 4.1. Further Interesting Findings 

Even though we could not confirm most of our hypotheses, our study provided some noteworthy findings. For example, we found weak evidence that the serial order effect is moderated by instruction type and workload. The analysis suggested that the be-fluent instruction leads to a stronger increase of creative quality across time. This finding may be due to the higher baseline of creative quality when the be-creative instruction is given: In the be-fluent instruction condition, participants start with very common uses, and when they run out of ideas they have to switch to unusual uses. Conversely, in the be-creative instruction condition, participants initially try to name unusual uses from the beginning, such that the potential for increasing creative quality over time is lower than in the be-fluent instruction condition. Furthermore, the analysis of the serial order effect suggested weak evidence that workload moderated the quadratic effect of time on creative quality. This effect was apparent through a decrease in creative quality at the end of the time period in the load condition. Perhaps this finding points to a cumulated load effect that inhibits participants from maintaining their level of creativity towards the end of the time period. Another interpretation might be that workload has a stronger impact at the end of the time period than the beginning, since executive processes are more strongly involved at the end than at the beginning of the task. The serial order findings in this work with respect to flexibility are also in accordance with this idea. Towards the end of the testing session for each of the tested conditions, it becomes very difficult to generate responses from categories that were not already used. That is, flexibility becomes less likely with increasing time-on-task and, hence, a shift from flexible thinking to persistence could explain why cognitive load also increasingly affects creative ideation (see Zhang et al. 2020). Although many studies have suggested that time is required for people to develop cognitive strategies that help them overcome dominant and already named ideas and make space for novel and more creative ideas (Beaty and Silvia 2012), we must consider that the results were ambiguous, and future studies are needed to allow for more reliable conclusions. 

Importantly, it should be noted that a direct comparison of the serial order effect between be-creative and be-fluent instruction conditions does not yet exist in the literature. The general shapes of the ideation functions (creative quality as a function of time; see Briggs and Reinig 2010) in all studied conditions is in accordance with a diminishing returns ideation function. This observation is also complemented by a negative correlation between fluency and creative quality. Overall, these results hint at a trade-off between quantity and quality in DT that was fairly robust across the studied conditions. Hence, this extends the equivocal picture of findings in the literature (see Forthmann et al. 2020a).

Another noteworthy result was the significant negative correlation between dose and quality in the load condition with the be-creative instruction. This finding suggests that the creative quality of ideas declines as more digits are presented. Since this correlation was not significant in the other instruction condition, it may be evidence (albeit weak) that cognitive load is involved in divergent thinking. This observation was further corroborated by revealing a similar pattern of findings for flexibility.

Our exploratory analysis revealed further findings that have yet to be explained. First, a higher dose led to higher accuracy in the be-creative instruction condition. Here, participants may have developed strategies during the task that made it easier for them to identify events. Yet, if this result appeared due to a training effect, it remains unclear why this correlation was only significant in the be-creative instruction condition. Second, we found a positive correlation between doses in the different instruction conditions that was independent of participants’ fluency. Therefore, the correlation is apparently due to another source of variance. 

### 4.2. Strengths and Limitations 

A clear strength of the present investigation is the experimental approach. Our study provides one of the first experimental designs to investigate a controlled component in creative thought. Although some methodological limitations exist, which are discussed subsequently, this approach may be used as starting point for future studies. A further strength of our study concerns the selection of task stimuli. We considered the frequency of objects to control for differences in the number of associations. Furthermore, we prevented carryover effects due to the semantic similarities between objects by constructing AUTs that contained objects from four different categories. Finally, the self-paced nature of the applied dual-task experiment is stronger in terms of external validity as compared to a procedure with a constantly demanding secondary task (e.g., Engström et al. 2005). That is, most situations outside the lab in which dual-task performance requires balancing of a creative thinking task and another task will most likely be self-paced.

However, we must consider that the latter strength (external validity) is also associated with the natural suspense between external and internal validity. In this vein, internal validity in this study was weakened, because the interval between two digits depended on the participant’s response latency for the presented object. Therefore, participants were not constantly exposed to the digit-tracking task. Furthermore, the number of digits to appear within the processing time of the AUTs was not defined. Instead, the number of presented digits increased along with the number of ideas a participant generated.^10^ This led to the following three problems: First, the workload was not equal in both instruction conditions. Since the number of generated ideas was higher in the be-fluent instruction condition than in the be-creative instruction condition, workload was higher in the be-fluent case. Second, contrary to our expectations, workload did not influence the performance on the experimental tasks. However, according to previous results (Rosen and Engle 1997), we would have expected that workload would at least impact fluency in the be-fluent instruction condition. Thus, our experimental design seemingly failed to produce a sufficient degree of workload. The third problem concerns the intended manipulation of executive functions. The purpose of our experimental design was to affect working memory in order to investigate the involvement of cognitive load in divergent thinking. However, administering working memory span tasks may affect the criterion validity of such tasks. For example, using a computerized version of the reading span task, Friedman and Miyake (2004) demonstrated that correlations between reading span scores and reading comprehension and verbal SAT scores^11^ were lower when participants were allowed to set the pace at which stimuli were presented compared to when an experimenter set the pace. Friedman and Miyake’s findings again highlight the trade-off between external and internal validity, and they concluded that a change of the administration method led to a change in criterion validity of the resulting measure (i.e., weakening internal validity also weakened criterion validity). In our study, even though participants could not influence how long a stimulus was presented, they were able to prolong the appearance of the following digit. Thus, in our study, the digit-tracking task may not have affected the participants’ working memory in the intended way.

These issues highlight that examining fluency in DT as the outcome in a self-paced (i.e., externally valid) dual-task paradigm might hardly be feasible. Creative quality and flexibility, however, can be examined by the correlational approach used in this work. That is, analyzing partial correlations between dose and creative quality with statistical control of fluency. But these analyses were not planned beforehand and, thus, sample size planning did not take this alternative strategy into account. Hence, we were only able to show that the amount of cognitive load was inversely linked with creative quality in the be-creative instruction condition, but statistical power to contrast this with the dose-quality correlation in the be-fluent instruction condition was most likely not sufficient. The same lines of argumentation apply to the analogous findings for flexibility.

A second methodological limitation of our study is the randomized presentation of objects during the AUTs (again an issue that might also been considered as a strength). Some participants reported a concurrent influx of several ideas for one object at one point (especially at the beginning of the task), which is compatible with the theory of the spreading activation network (Collins and Loftus 1975). They further complained that they had to memorize these ideas until the next time the object appeared. Following these important notes, it seems possible that the random presentation of objects in the AUTs created an unintended workload beyond our manipulation. 

Results concerning the validity of the experimental procedure were inconclusive. On the one hand, the replication of the be-creative effect (e.g., Nusbaum et al. 2014; Forthmann et al. 2016) and the serial order effect (Christensen et al. 1957; Beaty and Silvia 2012) can be seen as a validation of our experimental procedure, since these effects are typical findings in research on divergent thinking. On the other hand, the correlations between mean RIBS scores and the participants’ scores of creative quality were very weak, suggesting insufficient criterion validity. However, some other studies have reported modest correlations between RIBS scores and measures of divergent thinking (see, for example, Benedek et al. 2012b; An et al. 2016; Wilken et al. 2020). Furthermore, it has to be kept in mind that the RIBS is a self-report scale, which may go along with some shortcomings. For example, self-estimated creativity can be biased through social desirability (i.e., individuals may exaggerate their actual creative abilities to look better; Kaufman 2019) or the unawareness of one’s own creative potential (e.g., Kaufman and Beghetto 2013). 

### 4.3. Future Directions

Future studies should primarily address the methodological limitations that occurred in the present investigation. To increase internal validity, it would be meaningful to confront participants with a constant secondary workload that is independent of their fluency and their response latency. Rosen and Engle (1997), for example, used different modalities to simultaneously conduct the digit-tracking task and the category-generation task: They presented the digits visually and let participants generate members of categories verbally. However, this procedure required individually testing each participant. We opted for a more economic way of collecting data by testing groups of participants and letting them work at their own pace. In future work, an approach similar to that applied by Rosen and Engle (1997) that combines divergent thinking and working memory tasks might be more appropriate, even though it requires great effort. To also make the most out of the approach used in the current work (i.e., to keep external validity high), the correlational approach used here might be promising, but needs more careful planning.

Another point that needs further investigation concerns the validity of our experimental procedure. Particularly important would be investigating whether the randomized presentation of objects within an AUT produces an undesirable workload. Moreover, it would be interesting to analyze the impact of workload on the serial order effect. Our study provides weak evidence that the quadratic effect of time on creative quality is moderated through workload. Further investigation of this finding could give deeper insight into the process of idea generation. 

### 4.4. Conclusions

Despite of the methodological limitations of the present study, the idea of manipulating workload and instruction type as means to investigate the involvement of cognitive load in divergent thinking seems very promising. Further studies are needed to investigate controlled-attention theory using an experimental approach.

## Figures and Tables

**Table 1:** Means of fluency, creative quality, and flexibility scores separately for each factor level combination.

Instruction	Workload	Fluency	Quality	Flexibility
Be-fluent	No Load	49.314 (15.253)	1.949 (0.490)	0.303 (0.073)
	Load	49.608 (15.416)	1.915 (0.469)	0.312 (0.081)
Be-creative	No Load	28.726 (10.962)	2.931 (0.609)	0.485 (0.133)
	Load	30.314 (10.855)	2.927 (0.561)	0.457 (0.105)

Standard deviations are in parentheses. Quality = 75% quantile of participants’ creative quality.

**Table 2:** Analysis of the serial order effect on creative quality: Regression weights of fixed effects and model comparison statistics of the linear mixed-effects models.

Covariate	Model 1	Model 2	Model 3	Model 4	Model 5	Model 6	Model 7	Model 8	Model 9
	β (*SE*)	β (*SE*)	β (*SE*)	β (*SE*)	β (*SE*)	β (*SE*)	β (*SE*)	β (*SE*)	β (*SE*)
Intercept	1.448 (0.049) ***	1.445 (0.050) ***	1.448 (0.056) ***	2.000 (0.047) ***	2.072 (0.057) ***	1.991 (0.048) ***	1.997 (0.053) ***	2.070 (0.061) ***	2.084 (0.070) ***
Time	0.156 (0.013) ***	0.156 (0.013) ***	0.166 (0.018) ***	0.145 (0.011) ***	0.125 (0.018) ***	0.145 (0.011) ***	0.153 (0.015) ***	0.133 (0.021) ***	0.134 (0.025) ***
Time^2^	−0.010 (0.001) ***	−0.010 (0.001) ***	−0.011 (0.002) ***	−0.009 (0.001) ***	−0.008 (0.002) ***	−0.009 (0.001) ***	−0.010 (0.001) ***	−0.010 (0.002) ***	−0.010 (0.002) ***
Load		0.007 (0.017)	0.001 (0.055)			0.015 (0.015)	0.003 (0.048)	0.005 (0.048)	−0.023 (0.081)
Instruction				−0.817 (0.016) ***	−0.926 (0.050) ***	−0.817 (0.015) ***	−0.817 (0.016) ***	−0.925 (0.050) ***	−0.946 (0.071) ***
Time × load			−0.020 (0.025)				−0.017 (0.022)	−0.017 (0.022)	−0.020 (0.036)
Time^2^ × load			0.003 (0.002)				0.003 (0.002)	0.003 (0.002)	0.003 (0.003)
Time × instruction					0.029 (0.022)			0.028 (0.023)	0.026 (0.032)
Time^2^ × instruction					−0.001 (0.002)			−0.001 (0.002)	−0.001 (0.003)
Instruction × load									0.040 (0.100)
Time × load × instruction									0.006 (0.045)
Time^2^ × load × instruction									−0.001 (0.004)
AIC	19,109.92	19,111.74	19,108.32	16,802.46	16,794.39	16,803.43	16,798.43	16,790.45	16,794.80
BIC	19,144.89	19,153.70	19,164.27	16,844.42	16,850.35	16,852.39	16,861.38	16,867.39	16,892.72
$\Delta \chi^{2}$(*df*)	402.169 (2) ***^,a^	0.182 (1) ^b^	7.417 (2) *^,c^	2309.459 (1) ***^,d^	12.066 (2) **^,e^	1.029 (1) ^f^	8.998 (2) *^,g^	11.981 (2) **^,h^	1.649 (3) ^i^

Instruction is dummy coded with 0 = be-creative instruction and 1 = be-fluent instruction. Workload is dummy coded with 0 = load and 1 = no load. AIC = Akaike information criterion, BIC = Bayesian information criterion. ^a^ Model 1 tests linear and quadratic effects of time and was compared to a model that contained only an intercept. ^b^ Model 2 tests the effect of load and is compared with Model 1. ^c^ Model 3 tests the interaction effects of load and time, as well as of load and (time)^2^ and is compared with Model 2. ^d^ Model 4 tests the effect of instruction and is compared with Model 1. ^e^ Model 5 tests the interaction effects of instruction and time, as well as of instruction and (time)^2^ and is compared with Model 4. ^f^ Model 6 tests the effect of load and is compared with Model 4. ^g^ Model 7 tests the interaction effects of load and time, as well as of load and (time)^2^ and is compared with Model 6. ^h^ Model 8 tests the interaction effects of instruction and time, as well as of instruction and (time)^2^ and is compared with Model 7. ^i^ Model 9 tests the interaction effect of instruction and load, as well as the interaction effect of load, instruction and time and the interaction effect of load and instruction and (time)^2^ and is compared with Model 8. * *p* < 0.05; ** *p* < 0.01; *** *p* < 0.001.

**Table 3:** Analysis of the serial order effect on flexibility: Regression weights of fixed effects and model comparison statistics of the logistic linear mixed-effects models.

Covariate	Model 1	Model 2	Model 3	Model 4	Model 5	Model 6	Model 7	Model 8	Model 9
	β (*SE*)	β (*SE*)	β (*SE*)	β (*SE*)	β (*SE*)	β (*SE*)	β (*SE*)	β (*SE*)	β (*SE*)
Intercept	−1.086 (0.050) ***	−1.072 (0.056) ***	−1.060 (0.063) ***	−0.623 (0.060) ***	−0.590 (0.070) ***	−0.611 (0.065) ***	−0.602 (0.072) ***	−0.569 (0.081) ***	−0.603 (0.092) ***
Time	−0.700 (0.026) ***	−0.700 (0.026) ***	−0.721 (0.036) ***	−0.742 (0.027) ***	−0.887 (0.045) ***	−0.743 (0.027) ***	−0.764 (0.037) ***	−0.907 (0.052) ***	−0.908 (0.063) ***
Time^2^	0.431 (0.030) ***	0.431 (0.030) ***	0.417 (0.042) ***	0.452 (0.031) ***	0.425 (0.049) ***	0.452 (0.031) ***	0.440 (0.043) ***	0.413 (0.057) ***	0.411 (0.069) ***
Load		−0.030 (0.050)	−0.054 (0.080)			−0.023 (0.050)	−0.043 (0.082)	−0.044 (0.082)	0.027 (0.121)
Instruction				−0.804 (0.052) ***	−0.866 (0.082) ***	−0.804 (0.052) ***	−0.804 (0.052) ***	−0.866 (0.082) ***	−0.802 (0.115) ***
Time × load			0.041 (0.052)				0.043 (0.052)	0.041 (0.052)	0.044 (0.089)
Time^2^ × load			0.029 (0.060)				0.025 (0.061)	0.025 (0.061)	0.027 (0.097)
Time × instruction					0.238 (0.056) ***			0.238 (0.056) ***	0.246 (0.078) **
Time^2^ × instruction					0.076 (0.063)			0.076 (0.063)	0.073 (0.089)
Instruction × load									−0.132 (0.164)
Time × load × instruction									−0.018 (0.111)
Time^2^ × load × instruction									0.008 (0.126)
AIC	9588.79	9590.41	9593.26	9350.04	9328.13	9351.83	9354.74	9332.89	9337.46
BIC	9616.76	9625.39	9642.23	9385.01	9377.09	9393.80	9410.70	9402.83	9428.39
$\Delta \chi^{2}$(*df*)	843.31 (2) ***^,a^	0.37 (1) ^b^	1.15 (2) ^c^	240.75 (1) ***^,d^	25.92 (2) ***^,e^	0.21 (1) ^f^	1.09 (2) ^g^	25.85 (2) ***^,h^	1.43 (3) ^i^

Instruction is dummy coded with 0 = be-creative instruction and 1 = be-fluent instruction. Workload is dummy coded with 0 = load and 1 = no load. AIC = Akaike information criterion, BIC = Bayesian information criterion. ^a^ Model 1 tests linear and quadratic effects of time and was compared to a model that contained only an intercept. ^b^ Model 2 tests the effect of load and is compared with Model 1. ^c^ Model 3 tests the interaction effects of load and time, as well as of load and (time)^2^ and is compared with Model 2. ^d^ Model 4 tests the effect of instruction and is compared with Model 1. ^e^ Model 5 tests the interaction effects of instruction and time, as well as of instruction and (time)^2^, and is compared with Model 4. ^f^ Model 6 tests the effect of load and is compared with Model 4. ^g^ Model 7 tests the interaction effects of load and time, as well as of load and (time)^2^, and is compared with Model 6. ^h^ Model 8 tests the interaction effects of instruction and time, as well as of instruction and (time)^2^, and is compared with Model 7. ^i^ Model 9 tests the interaction effect of instruction and load, as well as the interaction effect of load, instruction and time, and the interaction effect of load and instruction and (time)^2^, and is compared with Model 8. * *p* < 0.05; ** *p* < 0.01; *** *p* < 0.001.

**Table 4:** Correlations of fluency and creative quality with dose, accuracy and self-reported ideational behavior for each separate experimental condition.

Measure	BF (No Load)			BF (Load)			BC (No Load)			BC (Load)		
	Fluency	Quality	Flexibility	Fluency	Quality	Flexibility	Fluency	Quality	Flexibility	Fluency	Quality	Flexibility
Fluency ^a^		−0.402	−0.481		−0.413	−0.484		−0.268	−0.698		−0.296	−0.623
Quality ^a^	−0.402		0.428	−0.413		0.449	−0.268			−0.296		0.470
Flexibility ^a^	−0.481	0.428		−0.484	0.449		−0.698	0.476		−0.623	0.470	
Dose BF	0.859	−0.389	−0.351	0.980	−0.446	−0.451	0.490	−0.097	−0.362	0.517	−0.072	−0.248
Dose BC	0.439	0.024	−0.294	0.520	−0.101	−0.325	0.666	−0.353	−0.581	0.975	−0.369	−0.651
Accuracy BF	0.150	−0.193	0.016	0.159	−0.159	−0.043	−0.061	0.037	0.067	0.016	−0.012	−0.029
Accuracy BC	0.116	−0.086	−0.086	0.242	−0.102	−0.171	0.175	−0.212	−0.384	0.319	−0.254	−0.491
RIBS	0.047	0.036	0.159	−0.026	0.080	0.439	−0.072	−0.110	0.137	0.077	−0.125	−0.107

BF = be-fluent instruction condition, BC = be-creative instruction condition. Quality = 75% quantile of a participant’s creative quality. RIBS = Runco Ideational Behavior Scale. ^a^ Correlations between fluency, quality, and flexibility were calculated within an experimental condition.

**Table 5:** Descriptive statistics and correlations between dose, accuracy, and self-reported ideational behavior.

Measure	M	SD	2.	3.	4.	5.
1. Dose BF	51.588	15.946	0.515 ***	0.173	0.274	−0.008
2. Dose BC	31.882	10.360		−0.008	0.357 *	0.099
3. Accuracy BF	0.781	0.153			0.222	0.149
4. Accuracy BC	0.752	0.257				0.082
5. RIBS ^a^	3.458	0.629				

BF = be-fluent instruction condition, BC = be-creative instruction condition. RIBS = Runco Ideational Behavior Scale. ^a^ Mean and standard deviation refer to the participants’ averaged RIBS scores (each item of the RIBS is measured on a scale ranging from 1 to 6). * *p* < 0.05; *** *p* < 0.001.

## Footnotes

1 The time limit was set to 10 min according to the procedure of Rosen and Engle (1997).

2 Working memory is considered to be one of the core executive functions (Diamond 2013). As the digit-tracking task used by Rosen and Engle (1997) affected functioning of working memory, it seemed to be an appropriate task for creating workload.

3 An event is defined by the occurrence of the third odd digit in a row appearing in the sequence. It is synonymous with the term “critical digits” used by Rosen and Engle (1997).

4 For the practice trials including an AUT, the objects knife and ball as well as stone and cup served as stimuli. These differed from the experimental task stimuli and were not controlled in terms of their lemma frequency. Furthermore, the be-fluent instruction was used for these AUTs.

5 In practice trials including digit tracking, 12 digits were presented whereby participants had to react on two events. A mistake could be made by either pressing the space bar in the absence of an event or by missing an event.

6 The ICC was calculated on the basis of the final dataset. Therefore, excluded ideas and ideas from excluded participants were not considered for the calculation. ICC scores, however, did only differ marginally from the unadjusted dataset.

7 Cohen’s *d* was calculated using the average standard deviation of both repeated measures (see for example Lakens 2013).

8 Workload affected fluency, but not in the intended manner (see Table 1).

9 The impact of workload on creative quality seemed to be greater in the be-fluent instruction condition than in the be-creative instruction condition (see Table 1). This finding contradicted our prediction.

10 This methodological inadequacy was also visible in the enormously high correlation between dose and fluency in the load conditions.

11 The SAT (=Scholastic Assessment Test) is a college admission test that is used in the United States of America.

## Appendix A

Table A1 includes all Alternate Uses Task stimuli used in this experiment along with the respective word frequency.

**Table A1:** Task stimuli and corresponding lemma frequencies.

Category	Object	Frequency
Tool	Shovel	0.59
	Axe	0.57
	Pliers	0.56
	Saw	0.45
Furniture	Table	2.14
	Bed	2.01
	Chair	1.70
	Cupboard	1.20
Vegetable	Tomato	0.78
	Pea	0.70
	Cucumber	0.64
	Pepper	0.45
Musical instrument	Drum	1.01
	Violin	0.85
	Trumpet	0.71
	Flute	0.65

Frequency = normalized log10-lemma frequency.

## Appendix B

Appendix B includes the general instruction for the experimental tasks:

Congratulations! You now have learned the two different procedures of experimental tasks.

Now the actual experiment may start!

The experiment consists of 4 experimental blocks in total. Each of these experimental blocks comprises **4 different objects** you should name uses for.

You will get a specific instruction before each experimental block, which comprises two elements:

1. In the first part of the instruction, you will find out if you will have to either name **as many uses as possible or as original/creative uses as possible** for the given objects (You will get an example which illustrates the task).

2. In the second part of the instruction, you will find out if you will either have to track digits simultaneously or not (“with digits” vs. “without digits”).

Click on “Continue” to start the first experimental block.

## Appendix C

Appendix C includes the instruction for the single AUT with the Be-Fluent Instruction (Experimental Block):

The following experimental block contains **4 objects** that will be presented to you over and over in random order.

(1) Please name **as many different uses as possible** for the given objects. In this task it is important for you to generate **many and different uses** for each object.

(2) The task format is “without digits”! Thus, you do not have to work on the digit- tracking task simultaneously!

You have **10 min** for this task!

Click on “Continue” to start the experimental block.

## Appendix D

Appendix D includes the instruction for the single AUT with the Be-Creative Instruction (Experimental Block):

The following experimental block contains **4 objects** that will be presented to you over and over in random order.

(1) Please name uses for the given objects which are **as unusual as possible**. Of course, there are usual ways to use these objects; in this task, however, it is important for you to mention all **unusual and creative uses** that spring to your mind.

(2) The task format is “without digits”! Thus, you do not have to work on the digit- tracking task simultaneously!

You have **10 min** for this task!

Click on “Continue” to start the experimental block.

## Appendix E

Appendix E includes the instruction for the AUT Combined with the Digit-Tracking Task and with the Be-Fluent Instruction (Experimental Block):

The following experimental block contains **4 objects** that will be presented to you over and over in random order.

(1) Please name **as many different uses as possible** for the given objects. In this task it is important for you to generate **many and different uses** for each object.

(2) The **task format is “with digits”**! Thus, you have to work on the **digit-tracking task simultaneously** which means that you have to press the **SPACE**-key if you observe **3 odd digits in a row**! Try to make as few mistakes as possible!

You have **10 min** for this task!

Click on “Continue” to start the experimental block.

## Appendix F

Appendix F includes the instruction for the AUT Combined with the Digit-Tracking Task and with Be-Creative Instruction (Experimental Block):

The following experimental block contains **4 objects** that will be presented to you over and over in random order.

(1) Please name uses for the given objects which are **as unusual as possible**. Of course, there are usual ways to use these objects; in this task, however, it is important for you to mention all **unusual and creative uses** that spring to your mind.

(2) The **task format is “with digits”**! Thus, you have to work on the **digit-tracking task simultaneously** which means that you have to press the **SPACE**-key if you observe **3 odd digits in a row**! Try to make as few mistakes as possible!

You have **10 min** for this task!

Click on “Continue” to start the experimental block.

## Appendix G

Appendix G includes the instruction for the Typing Speed Trial:

Before the experiment starts, we would like to measure your typing speed. In the following, your task will be to type the calendar months of a year in their typical sequence.

On each of the subsequent pages one textbox is at your disposal for typing in just one month.

Each time you enter a month you get to the next page by pressing the **ENTER**-key.

You start with the month “January”. If you arrive at “December”, please start from the beginning.

In order to make your start into this task easier, in the first two trials the months “January” and “February” will be displayed above the textboxes. Afterwards you are asked to continue the sequence without cues.

If you are uncertain which month goes next in the sequence during the task, you are allowed to start with “January” again.

It is sufficient in this task if you concentrate on the use of **lowercase letters**!

Please work as fast as you can. You have **60 s** to perform the task!

If you are ready, click on “Continue” to start the trial.

## Appendix H

Appendix H includes the general instruction for the Practice Trials:

In this experiment you will be asked to name either **many uses** or **original/creative uses** for particular objects.

However, first you need to become familiar with the different task formats. Thus, in the following you will work on a few practice trials.

There will be **two** different task formats you have to work on.

Click on “Continue” to discover the **first task format**!

## Appendix I

Appendix I includes the instruction for the first Practice Trial for Single AUT without Concurrent Digit-Tracking (First Task Format)

**First task format: “Without digits”**

In the following you will be presented an object in the middle of the computer screen.

Below the object there is a textbox in which you should type **one** use for the currently presented object.

If you named one use for the given object, press the **ENTER**-key to get to the next object.

There will be two different objects in this practice trial which are presented to you in a random order.

In this practice trial your task is to just name **as many uses as possible** for each object!

Click on “Continue” to start the practice trial.

## Appendix J

Appendix J includes the instruction for the Second Practice Trial for Single Digit-Tracking Task

Well done, you are familiar with the first task format now!

In the following you will discover the second task format which differs from the first task format in one important aspect:

During working on the previously introduced task you have to complete **another task** simultaneously.

The task is named **digit-tracking** and is constructed as follows:

You will be presented successively the digits from 1 to 9 in a random order. The first digit appears in the upper left corner. Subsequently, additional digits appear clockwise on the screen for the entire duration of the task block.

Thus, a long chain results consisting of digits ranging from 1 to 9!

Each digit is displayed to you for **1 s**!

Your task will be to press the **SPACE**-key if you observed **3 odd digits in a row**!

It does not matter in which corner the sequence starts!

Example:

3—7—3 → Press Space-key
5—9—4 → Don´t do anything

Click on “Continue” to practice this concurrent task **isolated**.

## Appendix K

Appendix K includes the instruction for the Third Practice Trial for the AUT Combined with the Digit-Tracking Task (Second Task Format)

**Second task format: “With digits”**

Since you have already discovered the first task format and the digit-tracking task, it will be just a small step to get familiar with the second task format!

The procedure is as follows:

As in the first task format, in the following practice trial you will be presented an object in the middle of the computer screen. Your task is to name one use for this object in the textbox below. Subsequently you have to press the **ENTER**-key to get to the next object.

**However, unlike the first task format a digit will appear on the screen for one second (as shown in the last practice trial) before the next object is being presented.**

Therefore, your task consists of two parts:

1. Name one use for each object that is presented to you.
2. Track the digits and press the SPACE-key if you observed 3 digits in a row.

In this practice trial your task is simply to name as **many** different uses for each object as possible!

Click on “Continue” to start the practice trial.

## Appendix L

Appendix L includes the running orders of experimental tasks.

Running order of experimental tasks depended on the identification number (ID) of each participant. There were eight different running orders that were assigned consecutively to the participants’ IDs. For example, the running order of experimental tasks was the same for participants with the IDs 1, 9, 17, 25, 33, 41 and 49.

In the following, the eight different running orders of experimental tasks are presented. For purposes of illustration the following abbreviations are used:

1 = Instruction: “be fluent”, Workload: no Load

2 = Instruction: “be creative”, Workload: no Load

3 = Instruction: “be fluent”, Workload: Load

4 = Instruction: “be creative”, Workload: Load

1st of each 8 participants: 1 → 2 → 3 → 4
2nd of each 8 participants: 3 → 4 → 1 → 2
3rd of each 8 participants: 1 → 2 → 4 → 3
4th of each 8 participants: 3 → 4 → 2 → 1
5th of each 8 participants: 2 → 1 → 3 → 4
6th of each 8 participants: 4 → 3 → 1 → 2
7th of each 8 participants: 2 → 1 → 4 → 3
8th of each 8 participants: 4 → 3 → 2 → 1

## References

[B1-jintelligence-09-00003] Acar Selcuk, Runco Mark A. (2017). Latency predicts category switch in divergent thinking. Psychology of Aesthetics, Creativity, and the Arts.

[B2-jintelligence-09-00003] Acar Selcuk, Runco Mark A., Ogurlu Uzeyir (2019). The moderating influence of idea sequence: A re-analysis of the relationship between category switch and latency. Personality and Individual Differences.

[B3-jintelligence-09-00003] Alvarez Julie A., Emory Eugene (2006). Executive function and the frontal lobes: A meta-analytic review. Neuropsychology Review.

[B4-jintelligence-09-00003] An Donggun, Song Youngmyung, Carr Martha (2016). A comparison of two models of creativity: Divergent thinking and creative expert performance. Personality and Individual Differences.

[B5-jintelligence-09-00003] Bates Douglas, Mächler Martin, Bolker Ben, Walker Steve (2015). Fitting linear mixed-effects models using lme4. Journal of Statistical Software.

[B6-jintelligence-09-00003] Beaty Roger E., Silvia Paul J. (2012). Why do ideas get more creative across time? An executive interpretation of the serial order effect in divergent thinking tasks. Psychology of Aesthetics, Creativity, and the Arts.

[B7-jintelligence-09-00003] Beaty Roger E., Silvia Paul J., Nusbaum Emily C., Jauk Emanuel, Benedek Mathias (2014). The roles of associative and executive processes in creative cognition. Memory & Cognition.

[B8-jintelligence-09-00003] Beaty Roger E., Kenett Yoed N., Hass Rick W., Shacter Danile L. (2019). A fan effect for creative thought: Semantic richness facilitates idea quantity but constrains idea quality. PsyArXiv.

[B9-jintelligence-09-00003] Benedek Mathias, Franz Fabiola, Heene Moritz, Neubauer Aljoscha C. (2012a). Differential effects of cognitive inhibition and intelligence on creativity. Personality and Individual Differences.

[B10-jintelligence-09-00003] Benedek Mathias, Könen Tanja, Neubauer Aljoscha C. (2012b). Associative abilities underlying creativity. Psychology of Aesthetics, Creativity, and the Arts.

[B11-jintelligence-09-00003] Benedek Mathias, Jauk Emanuel, Fink Andreas, Koschutnig Karl, Reishofer Gernot, Ebner Franz, Neubauer Aljoscha C. (2014a). To create or to recall? Neural mechanisms underlying the generation of creative new ideas. NeuroImage.

[B12-jintelligence-09-00003] Benedek Mathias, Jauk Emanuel, Sommer Markus, Arendasy Martin, Neubauer Aljoscha C. (2014b). Intelligence, creativity, and cognitive control: The common and differential involvement of executive functions in intelligence and creativity. Intelligence.

[B13-jintelligence-09-00003] Bien Heidrun, Levelt Willem J. M., Baayen R. Harald (2005). Frequency effects in compound production. Proceedings of the National Academy of Sciences of the United States of America.

[B14-jintelligence-09-00003] Briggs Robert O., Reinig Bruce A. (2010). Bounded ideation theory. Journal of Management Information Systems.

[B15-jintelligence-09-00003] Brophy Dennis R. (1998). Understanding, measuring, and enhancing individual creative problem-solving efforts. Creativity Research Journal.

[B16-jintelligence-09-00003] Carroll John B. (1993). Human Cognitive Abilities: A Survey of Factor-Analytic Studies.

[B17-jintelligence-09-00003] Christensen Paul R., Guilford Joy P., Wilson Robert C. (1957). Relations of creative responses to working time and instructions. Journal of Experimental Psychology.

[B18-jintelligence-09-00003] Collins Allan M., Loftus Elizabeth F. (1975). A spreading-activation theory of semantic processing. Psychological Review.

[B19-jintelligence-09-00003] Cropley Arthur (2006). In praise of convergent thinking. Creativity Research Journal.

[B20-jintelligence-09-00003] Diamond Adele (2013). Executive functions. Annual Review of Psychology.

[B21-jintelligence-09-00003] Engström Johan, Johansson Emma, Östlund Joakim (2005). Effects of visual and cognitive load in real and simulated motorway driving. Transportation Research Part F.

[B22-jintelligence-09-00003] Forthmann Boris, Gerwig Anne, Holling Heinz, Çelik Pinar, Storme Martin, Lubart Todd (2016). The be-creative effect in divergent thinking: The interplay of instruction and object frequency. Intelligence.

[B23-jintelligence-09-00003] Forthmann Boris, Holling Heinz, Zandi Nima, Gerwig Anne, Çelik Pinar, Storme Martin, Lubart Todd (2017a). Missing creativity: The effect of cognitive workload on rater (dis-)agreement in subjective divergent-thinking scores. Thinking Skills and Creativity.

[B24-jintelligence-09-00003] Forthmann Boris, Holling Heinz, Çelik Pinar, Storme Martin, Lubart Todd (2017b). Typing speed as a confounding variable and the measurement of quality in divergent thinking. Creativity Research Journal.

[B25-jintelligence-09-00003] Forthmann Boris, Regehr Sandra, Seidel Julia, Holling Heinz, Çelik Pinar, Storme Martin, Lubart Todd (2018). Revisiting the interactive effect of multicultural experience and openness to experience on divergent thinking. International Journal of Intercultural Relations.

[B26-jintelligence-09-00003] Forthmann Boris, Szardenings Carsten, Holling Heinz (2020a). Understanding the confounding effect of fluency in divergent thinking scores: Revisiting average scores to quantify artifactual correlation. Psychology of Aesthetics, Creativity, and the Arts.

[B27-jintelligence-09-00003] Forthmann Boris, Paek Sue H., Dumas Denis, Barbot Baptiste, Holling Heinz (2020b). Scrutinizing the basis of originality in divergent thinking tests: On the measurement precision of response propensity estimates. British Journal of Educational Psychology.

[B28-jintelligence-09-00003] Friedman Naomi P., Miyake Akira (2004). The reading span test and its predictive power for reading comprehension ability. Journal of Memory & Language.

[B29-jintelligence-09-00003] Gabora Liane (2010). Revenge of the “Neurds”: Characterizing creative thought in terms of the structure and dynamics of memory. Creativity Research Journal.

[B30-jintelligence-09-00003] GenABEL Project Developers (2013). GenABEL: Genome-Wide Snp Association Analysis. R package Version 1.8-0. http://CRAN.R-project.org/package=GenABEL.

[B31-jintelligence-09-00003] Gilhooly Kenneth J., Fioratou Evridiki, Anthony Susan H., Wynn Val (2007). Divergent thinking: Strategies and executive involvement in generating novel uses for familiar objects. British Journal of Psychology.

[B32-jintelligence-09-00003] Guilford Joy Paul (1968). Intelligence, Creativity, and Their Educational Implications.

[B33-jintelligence-09-00003] Harrington David M. (1975). Effects of explicit instructions to “be creative” on the psychological meaning of divergent thinking test scores. Journal of Personality.

[B34-jintelligence-09-00003] Hommel Bernhard (2015). Between persistence and flexibility: The Yin and Yang of action control. Advances in Motivation Science.

[B35-jintelligence-09-00003] Kaufman James C. (2019). Self-assessments of creativity: Not ideal, but better than you think. Psychology of Aesthetics, Creativity, and the Arts.

[B36-jintelligence-09-00003] Kaufman James C., Beghetto Ron A. (2013). In parise of Clark Kent: Creativity metacognition and the importance of teaching kids when (not) to be creative. Roeper Review.

[B37-jintelligence-09-00003] Kenett Yoed N., Anaki David, Faust Miriam (2014). Investigating the structure of semantic networks in low and high creative persons. Frontiers in Human Neuroscience.

[B38-jintelligence-09-00003] Kim Kyung Hee (2005). Can only intelligent people be creative?. Journal of Secondary Gifted Education.

[B39-jintelligence-09-00003] Lakens Daniël (2013). Calculating and reporting effect sizes to facilitate cumulative science: A practical primer for *t*-tests and ANOVAs. Frontiers in Psychology.

[B40-jintelligence-09-00003] Lawrence Michael A. (2013). ez: Easy Analysis and Visualization of Factorial Experiments. R package Version 4.2-2. http://CRAN.R-project.org/package=ez.

[B41-jintelligence-09-00003] Lubart Todd I. (2001). Models of the creative process: Past, present and future. Creativity Research Journal.

[B42-jintelligence-09-00003] Mednick Sarnoff (1962). The associative basis of the creative process. Psychological Review.

[B43-jintelligence-09-00003] Mumford Michael, McIntosh Tristan (2017). Creative thinking processes: The past and the future. Journal of Creative Behavior.

[B44-jintelligence-09-00003] Nijstad Bernard A., De Dreu Carsten K. W., Rietzschel Eric F., Baas Matthijs (2010). The dual pathway to creativity model: Creative ideation as a function of flexibility and persistence. European Review of Social Psychology.

[B45-jintelligence-09-00003] Nusbaum Emily C., Silvia Paul J. (2011). Are intelligence and creativity really so different? Fluid intelligence, executive processes, and strategy use in divergent thinking. Intelligence.

[B46-jintelligence-09-00003] Nusbaum Emily C., Silvia Paul J., Beaty Roger E. (2014). Ready, set, create: What instructing people to “Be creative” reveals about the meaning and mechanisms of divergent thinking. Psychology of Aesthetics, Creativity, and the Arts.

[B47-jintelligence-09-00003] Pan Xuan, Yu Huihong (2016). Different effects of cognitive shifting and intelligence on creativity. Journal of Creative Behavior.

[B48-jintelligence-09-00003] Pinheiro Jose, Bates Douglas, DebRoy Saikat, Sarkar Deepayan, R Core Team (2015). nlme: Linear and Nonlinear Mixed Effects Models. R package Version 3.1-120. http://CRAN.R-project.org/package=nlme.

[B49-jintelligence-09-00003] R Core Team (2014). R: A Language and Environment for Statistical Computing.

[B50-jintelligence-09-00003] Reiter-Palmon Roni, Forthmann Boris, Barbot Baptiste (2019). Scoring divergent thinking tests: A review and systematic framework. Psychology of Aesthetics, Creativity, and the Arts.

[B51-jintelligence-09-00003] Rosen Virginia M., Engle Randall W. (1997). The role of working memory capacity in retrieval. Journal of Experimental Psychology: General.

[B52-jintelligence-09-00003] Runco Mark A., Acar Selcuk (2010). Do tests of divergent thinking have an experiential bias?. Psychology of Aesthetics, Creativity, and the Arts.

[B53-jintelligence-09-00003] Runco Mark A., Acar Selcuk (2012). Divergent thinking as an indicator of creative potential. Creativity Research Journal.

[B54-jintelligence-09-00003] Runco Mark A., Okuda Shawn M. (1991). The instructional enhancement of the flexibility and originality scores of divergent thinking tests. Applied Cognitive Psychology.

[B55-jintelligence-09-00003] Runco Mark A., Plucker Jonathan A., Lim Woong (2001). Development and psychometric integrity of a measure of ideational behavior. Creativity Research Journal.

[B56-jintelligence-09-00003] Schröder Astrid, Gemballa Teresa, Ruppin Steffie, Wartenburger Isabell (2012). German norms for semantic typicality, age of acquisition, and concept familiarity. Behavior Research Methods.

[B57-jintelligence-09-00003] Silvia Paul J. (2008). Another look at creativity and intelligence: Exploring higher-order models and probable confounds. Personality and Individual differences.

[B58-jintelligence-09-00003] Silvia Paul J. (2015). Intelligence and creativity are pretty similar after all. Educational Psychology Review.

[B59-jintelligence-09-00003] Silvia Paul J., Winterstein Beate P., Willse John T., Barona Christopher M., Cram Joshua T., Hess Karl I., Martinez Jenna L., Richard Crystal A. (2008). Assessing creativity with divergent thinking tasks: Exploring the reliability and validity of new subjective scoring methods. Psychology of Aesthetics, Creativity, and the Arts.

[B60-jintelligence-09-00003] Silvia Paul J., Martin Christopher, Nusbaum Emily C. (2009). A snapshot of creativity: Evaluating a quick and simple method for assessing divergent thinking. Thinking Skills and Creativity.

[B61-jintelligence-09-00003] Silvia Paul J., Beaty Roger E., Nusbaum Emily C. (2013). Verbal fluency and creativity: General and specific contributions of broad retrieval ability (Gr) factors to divergent thinking. Intelligence.

[B62-jintelligence-09-00003] Steiger James H. (1980). Tests for comparing elements of a correlation matrix. Psychological Bulletin.

[B63-jintelligence-09-00003] Süß Heinz-Martin, Oberauer Klaus, Wittmann Werner W., Wilhelm Oliver, Schulze Ralf (2002). Working memory capacity explains reasoning ability—And a little bit more. Intelligence.

[B64-jintelligence-09-00003] Wallach Michael A., Kogan Nathan (1965). Modes of Thinking in Young Children: A Study of the Creativity-Intelligence Distinction.

[B65-jintelligence-09-00003] Wilken Andrea, Forthmann Boris, Holling Heinz (2020). Instructions moderate the relationship between creative performance in figural divergent thinking and reasoning capacity. Journal of Creative Behavior.

[B66-jintelligence-09-00003] Wilson Robert C., Guilford Joy P., Christensen Paul R. (1953). The measurement of individual differences in originality. Psychological Bulletin.

[B67-jintelligence-09-00003] Zabelina Darya, Jung Rex E., Vartanian Oshin (2018). Attention and creativity. The Cambridge Handbook of the Neuroscience of Creativity.

[B68-jintelligence-09-00003] Zabelina Darya, Saporta Arielle, Beeman Mark (2016a). Flexible or leaky attention in creative people? Distinct patterns of attention for different types of creative thinking. Memory & Cognition.

[B69-jintelligence-09-00003] Zabelina Darya, Colzato Lorenza, Beeman Mark, Hommel Bernhard (2016b). Dopamine and the creative mind: Individual differences in creativity are predicted by interactions between dopamine genes DAT and COMT. PLoS ONE.

[B70-jintelligence-09-00003] Zhang Weitao, Sjoerds Zsuzsika, Hommel Bernhard (2020). Metacontrol of human creativity: The neurocognitive mechanisms of convergent and divergent thinking. NeuroImage.

